# Mutations in human prion-like domains: pathogenic but not always amyloidogenic

**DOI:** 10.1080/19336896.2024.2329186

**Published:** 2024-03-21

**Authors:** Andrea Bartolomé-Nafría, Javier García-Pardo, Salvador Ventura

**Affiliations:** Institut de Biotecnologia i de Biomedicina (IBB) and Departament de Bioquímica i Biologia Molecular, Universitat Autònoma de Barcelona, Barcelona, Spain

**Keywords:** Amyloid, Cryo-EM structure, functional amyloids, low complexity domain, Neurodegeneration, Ribonucleoprotein, RNA-binding protein

## Abstract

Heterogeneous nuclear ribonucleoproteins (hnRNPs) are multifunctional proteins with integral roles in RNA metabolism and the regulation of alternative splicing. These proteins typically contain prion-like domains of low complexity (PrLDs or LCDs) that govern their assembly into either functional or pathological amyloid fibrils. To date, over 60 mutations targeting the LCDs of hnRNPs have been identified and associated with a spectrum of neurodegenerative diseases including amyotrophic lateral sclerosis (ALS), frontotemporal dementia (FTD), and Alzheimer’s disease (AD). The cryo-EM structures of pathological and functional fibrils formed by different hnRNPs have been recently elucidated, including those of hnRNPA1, hnRNPA2, hnRNPDL-2, TDP-43, and FUS. In this review, we discuss the structural features of these amyloid assemblies, placing particular emphasis on scrutinizing the impact of prevalent disease-associated mutations mapping within their LCDs. By performing systematic energy calculations, we reveal a prevailing trend of destabilizing effects induced by these mutations in the amyloid structure, challenging the traditionally assumed correlation between pathogenicity and amyloidogenic propensity. Understanding the molecular basis of this discrepancy might provide insights for developing targeted therapeutic strategies to combat hnRNP-associated diseases.

## Introduction

Amyloid fibrils are highly ordered supramolecular aggregates, where identical protein molecules stack in a fibrillar conformation. They typically exhibit a cross-ß fold, where ß-sheets are often twisted slightly from one layer to the next, creating a twisting helix along the fibril’s axis [1,2]. Steric zippers are prevalent in amyloid fibrils, effectively sealing off the structure from solvent access, contributing to its remarkable stability [3]. Interestingly, many of the structured regions in amyloids arise from intrinsically disordered regions (IDRs) of low complexity, known as low complexity domains (LCDs), in their soluble counterparts. These regions are key in facilitating self-templated aggregation, a process well-documented in yeast prions and human prion-like domains (PrLDs) [4–6].

For many years, amyloid aggregates have been primarily associated with pathology, linked to a range of debilitating diseases such as Alzheimer’s disease (AD), Parkinson’s disease (PD), and type II diabetes [2]. However, a growing body of research has revealed that amyloids can also serve crucial biological functions across all domains of life [1,2]. For instance, amyloids have been shown to participate in structural support, storage, transmission of information or signalling [2]. However, under certain conditions, such as increased protein concentration or genetic mutations, even these functional amyloids can undergo a transformation into more stable, irreversible, and pathological forms.

A significant fraction of the proteins shown to transition to amyloids have RNA-processing functions, with a notable representation of RNA-binding proteins (RBPs) [1,7,8]. RBPs constitute a large class of proteins that, together with RNA, form complexes within the nucleus, known as ribonucleoproteins (RNPs). RNPs are crucial in RNA metabolism, especially in cells with complex and dynamic RNA profiles like neurons or glial cells [7–11]. Among them, we find heterogeneous nuclear ribonucleoproteins (hnRNPs), which are involved in all aspects of RNA metabolism and in the regulation of alternative splicing [12]. They exhibit a modular structure, containing one or more RNA binding domains (RBDs) that mediate interactions with nucleic acids, the most common being the RNA recognition motif (RRM). Additionally, they possess at least one LCD, which is required for establishing relevant protein-protein interactions [4,7,9,13]. The LCD often, but not always, includes the nuclear localization sequence (NLS), facilitating nucleocytoplasmic shuttling as needed [9]. Recent evidence suggests that the activity of hnRNPs is strongly associated to their ability to switch between monomeric to amyloid-like conformations, modulated by their LCDs, and eventually influenced by the NLS [10].

**Figure 1.**
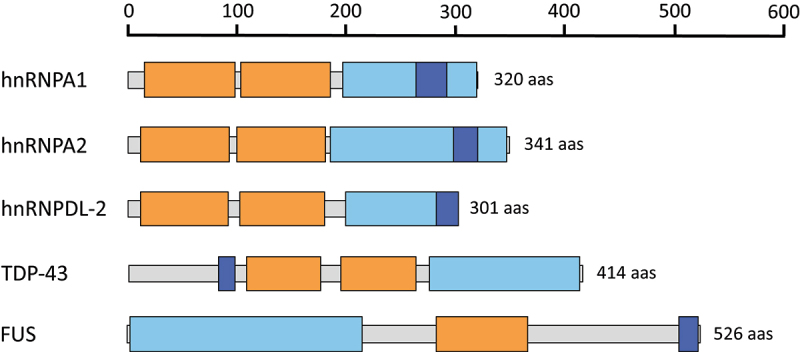
Domain organization of hnRNPs that have been structurally characterized using cryo-EM. The RNA recognition motifs (RRMs), low complexity domains (LCDs) and the nuclear localization signals (PY-NLS and NLS) are colored in orange, light blue and dark blue, respectively.

Recent advancements in cryo-electron microscopy (cryo-EM) technology have enabled the determination of the atomic structures of diverse hnRNP assemblies [14–16]. The high-resolution structures of the amyloid fibrils formed by the LCDs of hnRNPA1 (PDB 7BX7) [17], hnRNPA2 (PDB 6WQK, PDB 8DU2, PDB 8DUW, PDB 8EC7) [18,19], TAR DNA-binding protein 43 (TDP-43) (PDB 7Q3U, PDB 7KWZ, PDB 6N37, PDB 6N3B, PDB 6N3A, PDB 6N3C) [20–22] and Fused in sarcoma (FUS) (PDB 6XFM, PDB 7VQQ) [23,24] have been already reported (Figure 1). Additional studies have also unveiled the structure of the amyloid fibrils formed by full-length hnRNPs, including hnRNPDL-2 (PDB 7ZIR) [25], hnRNPA1 (PDB 7ZJ2) [26] and TDP-43 (PDB 7PY2, PDB 8CG3) [27,28] (Figure 1). Taken together, these studies have shown that while most pathologic amyloids can adopt multiple highly stable fibril structures, known as polymorphs, functional amyloids typically adopt a single favourable conformational state, exhibiting more polar amyloid cores and a wider range of stabilities. This confers lability and reversibility to the fibrils when they are no longer advantageous.

More than 60 mutations in the LCDs of hnRNPs have been linked to human diseases, including missense mutations in the hnRNPA1 [17,29,30], hnRNPA2 [4,18,31,32], TDP-43 [33–37], FUS [38,39] and hnRNPDL [10,25] genes. They have been traditionally thought to cause a gain of toxic function giving rise to different human disorders, including amyotrophic lateral sclerosis (ALS), frontotemporal dementia (FTD), multisystem proteinopathy (MSP), Alzheimer’s disease (AD) or Parkinson’s disease (PD) [1,9,13]. A fraction of these mutations target the NLS motif of these proteins, resulting in cytoplasmic mislocalization and the accumulation of persistent proteinaceous inclusions [29]. Interestingly, emerging research suggests that certain missense mutations in hnRNPs do not favour fibrillation, as was often assumed. A notable example is hnRNPDL-2, an RBP involved in transcription and RNA processing, where mutations have been linked to limb-girdle muscular dystrophy D3 (LGMD D3). These mutations are associated with a reduced tendency to fibrillate, correlating with the absence of protein aggregates in the muscular tissues of most LGMD D3 affected patients [10,25].

Understanding how LCD mutations in hnRNPs influence fibril formation and regulate structure-function relationships is essential for elucidating the genetic basis of these diseases. In this review, we provide a comprehensive overview of the current structural understanding of hnRNP assemblies, focusing on how disease-associated mutations affect their structural and functional integrity.

### Disease-associated mutations in hnRNPA1 and hnRNPA2

**Figure 2.**
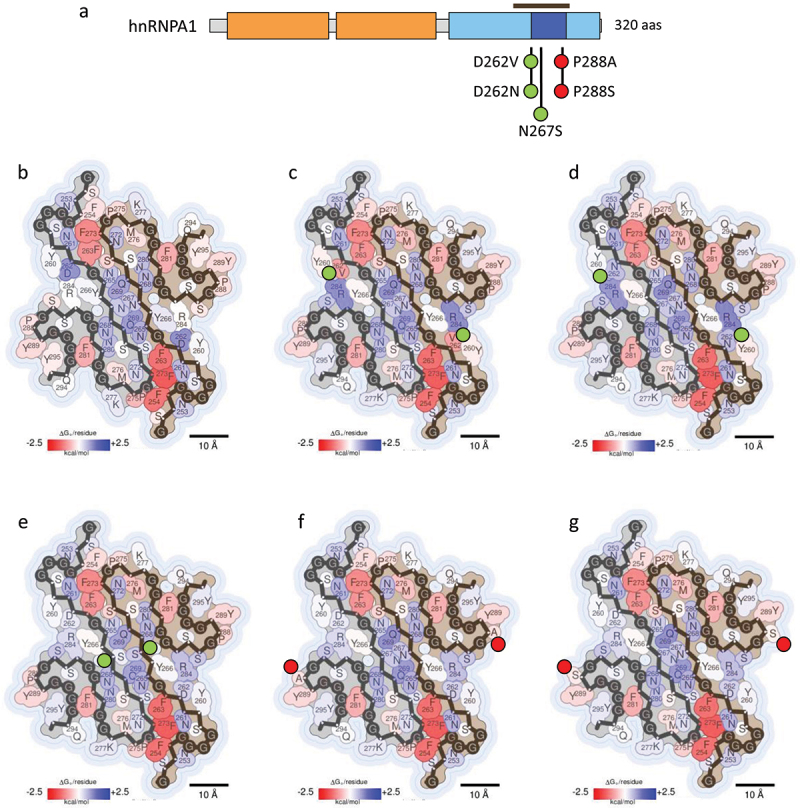
Impact of hnRNPA1 disease-associated mutations on fibril stability. (a) domain organization of hnRNPA1. Residues covered by the amyloid core in the cryo-EM hnRNPA1 fibril structure (PDB 7BX7) [17] are indicated with a brown bar. (b-g) stabilization energy maps of the amyloid fibrils formed by the WT protein (b) and their corresponding disease-associated mutants D262V (c), D262N (d), N267S (e), P288A (f) and P288S (g). Note that amyloid fibril structures are colored by energy, being red and blue the stabilizing and destabilizing residues, respectively. In (a-g) the nature of the disease-associated mutations is indicated with green (stabilizing) or red (destabilizing) filled circles.

**Figure 3.**
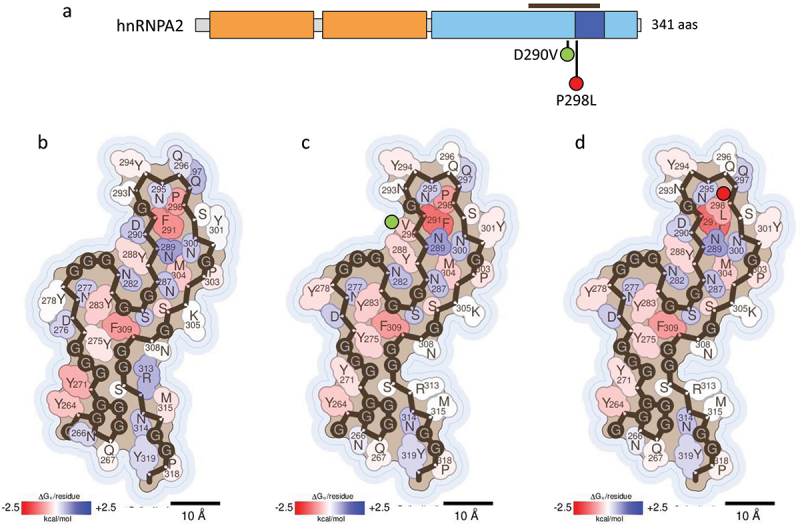
Impact of hnRNPA2 disease-associated mutations on fibril stability. (a) domain organization of hnRNPA2. Residues covered by the amyloid core in the cryo-EM hnRNPA2 fibril structure (PDB 6WQK) [18] are indicated with a brown bar. (b-d) stabilization energy maps of the amyloid fibrils formed by the WT (b) and their corresponding disease-associated mutants D290V (c) and P298L (d). The structures are colored according to the energy values, as described in figure 2. In (a-d), the nature of the disease-associated mutations is indicated with green (stabilizing) or red (destabilizing) filled circles.

hnRNPA1 and hnRNPA2 (A2) are integral components of the 40S ribonucleoprotein (RNP) complex, a key player in nuclear RNA packaging [10]. Notably, three isoforms of hnRNPA1 are produced by alternative splicing, being hnRNPA1A (hereafter referred as A1) the predominant variant [40] and the isoform described in this review. Both A1 and A2 proteins are highly expressed in cells and share a similar domain architecture consisting of two RRMs and a LCD that includes the Proline-Tyrosine nuclear localization sequence (PY-NLS) Figure 2(a) and Figure 3(a) [9,10]. These proteins have been described to undergo liquid-liquid phase separation (LLPS) and self-assemble into both pathological and functional fibrils [17,18,31,41].

Recently, the cryo-EM structures of the amyloid fibrils formed by A1 and A2 LCD, have been determined [17,18]. These high-resolution structures revealed that the filament amyloid cores of A1 and A2 encompass their PY-NLS sequences. Furthermore, in line with their reversible nature, A1 and A2 fibrils display greater lability compared to other pathogenic amyloid assemblies formed by hnRNPs [17,18,30]. In this scenario, disease-associated mutations in A1 and A2 LCDs were predicted to alter the dynamics of these proteins in the cell promoting their transition into ordered aggregated structures that can interfere with normal cellular processes, contributing to the development of degenerative diseases [4,9]. To investigate the impact of these disease-associated mutations, we systematically computed the energy changes caused by these amino acid substitutions in the amyloid assembly using Rosetta (Table 1).

**Table 1.** Calculated standard free energy of stabilization of disease-associated mutations in hnRNPs.

	Mutation	Related disease	ΔΔG (kcal/mol)*	References
hnRNPA1 (PDB 7BX7) [17]	D262V	ALS, MSP	−24,19	[17,29,30]
	D262N	ALS, MSP	−11,32	[17,29,30]
	P288S	ALS, MSP	34,08	[17,29]
	P288A	ALS, MSP	15,01	[17,29]
	N267S	ALS, MSP	−5,25	[17,29]
hnRNPA2 (PDB 6WQK) [18]	D290V	MSP, ALS, Paget’s disease of bone	−2,77	[4,18,31]
	P298L	Paget’s disease of bone	12,07	[31,32]
hnRNPDL-2 (PDB 7ZIR) [25]	D259N	LGMD D3	−4,63	[10,25]
	D259H	LGMD D3	4,89	[10,25]
TDP-43 (PDB 7PY2) [27]	A315E	ALS	3,25	[33–35]
	A315T	ALS	4,75	[34–36]
	M337V	ALS	−5,29	[33,35,37]
	Q331K	ALS	14,48	[33,35,37]
TDP-43 (PDB 8CG3) [28]	A315E	ALS	4,83	[33–35]
	A315T	ALS	14,21	[34–36]
	M337V	ALS	31,86	[33,35,37]
	Q331K	ALS	22,93	[33,35,37]
FUS (PDB 7VQQ) [24]	P106L	FTLD	17,72	[38]
	S115N	ALS	−7,58	[39]

*Standard free energy of stabilization (ΔΔG in kcal/mol) upon mutation calculated using Rosetta [42] and the indicated cryo-EM structures. The mutations were classified as stabilizing or destabilizing, considering a threshold of ΔΔG <-1 kcal/mol for the first and ΔΔG >1 kcal/mol for the latest. Calculations were performed as described elsewhere [25].

In the case of A1, a set of single-point mutations within its LCD, including the missense mutations D262N/V, N267S, and P288S/A, have been linked to the development of ALS, MSP, and FTD [10,17,25,29] (Figure 2). Notably, the residues N267 and P288 are located within the PY-NLS region of the A1 LCD, playing a critical role in binding to the nuclear import receptor karyopherin-ß2 (Kapβ2). This binding is essential not only for the nuclear import of A1 but also for preventing its amyloid fibrillation, the receptor potentially acting as a chaperone to dissolve preformed fibrils. On the contrary, D262N/V mutation is located outside PY-NLS, being less likely to alter cellular localization of A1.

Energetic calculations assessing the impact of these mutations on the amyloid structure of A1 LCD (PDB 7BX7) [17] Table 1 and Figure 2(b-g), revealed an increased stability for the D262N/V and N267S mutations Figure 2(c-e), potentially enhancing their aggregation tendencies by facilitating the establishment of additional or stronger contacts. D262N/V mutant proteins have been reported to yield fibrils with a similar morphology to that of the WT protein [17], but these fibrils were reported to be more stable, consistent with Sun *et al*. and Gui *et al*. experiments, in which Asp mutations impaired fibril reversibility as measured by proteinase K digestion or increasing the temperature, respectively [17,30]. Moreover, Beijer *et al*. and Sharma *et al*. reported that although D262V does not notably affect LLPS properties of the A1 mutant protein, it significantly delays stress granules (SGs) disassembly *in vitro*, supporting these previous findings [26,29]. In a similar manner, the N267S mutation has been reported to introduce a steric zipper that stabilizes the A1 protofilament interface [4,17]. Additionally, this mutation may also have an impact on Kapß2 binding, thus impairing Kapß2-mediated chaperone activity.

On the other hand, P288S/A Figure 2(f-g) effects might not seem to be obvious considering only the current structural context, since these mutations were predicted to be highly destabilizing. Although P288 appears to be oriented away from the fibril core in the WT structure, this residue participates in the Kapβ2 binding. Overall, P288A LLPS properties are similar to those of the WT protein, but it delays SG disassembly *in vitro*, similar to the D262V mutation [29]. Moreover, both P288S/A mutants accelerate A1 fibrillation [26,29], a result that is consistent with ZipperDB showing an introduction of a new steric zipper upon mutation [29]. Thus, P288S/A effect might be driven by a reduced Kapß2 binding, allowing the PY-NLS to engage in novel amyloid interactions and accumulation of A1 in the cytoplasm. However, energetic calculations suggest that the fine structure of the mutant fibrils would differ from that of the WT protein. A recent study has reported the architecture of the fibrils formed by full-length hnRNPA1 (PDB 7ZJ2) [26]. The core of these fibrils corresponds to the protein LCD and its structural configuration mirrors that of the fibrils formed by the LCD alone. Accordingly, computational analyses suggest that mutations exert a comparable thermodynamic influence on the integrity of these full-length fibril structures.

Point mutations in the A2 gene have also been implicated in the development of ALS and MSP (Figure 3). The D290V substitution bears striking similarities to the D262V mutation identified in A1 [4,18,31]. Fibril stability calculations using the A2 structure (PDB 6WQK) [18] Figure 3(b-d) reveals that this mutation confers greater thermodynamic stability to the fibrils Table 1 and Figure 3(c). This finding aligns with the work of Lu and colleagues [18], who reported the first cryo-EM structure of the amyloid fibrils formed by the LCD of this protein. They also crystallized a hexameric segment containing the D290V substitution [18]. Upon comparing the WT and mutant crystal structures, they observed the formation of steric zippers in the D290V variant, which may shift reversible into irreversible fibril aggregation [10,18].

In a recent study, the same research group elucidated the cryo-EM structures of three polymorphs generated by the D290V mutation in the LCD of A2, with findings consistent with our calculations, since the resulting fibrils were more stable than that of the WT protein [19]. Additionally, other studies have shown that D290V enhances A2 recruitment within cytoplasmatic SGs and induces aggregation [4,31,43,44]. Therefore, the pathogenicity of the D290V mutant might be attributed to both enhanced aggregation and the mislocalization and accumulation of irreversible A2 aggregates within the cytoplasm.

A second mutation in the LCD of A2, specifically P298L, has been associated with development of Paget’s disease of bone [31,44]. Some studies suggest that proteins harbouring the P298L mutation exhibit altered LLPS behaviour and increased aggregation propensity *in vitro*. [44] Although the exact effects of the P298L mutation on A2 are not fully understood, our calculations indicate that it may lead to decreased fibrils stability Table 1 and Figure 3(d). As P298 is located within the PY-NLS, its pathologic effects might be by an impaired binding of Kapß2 to the A2 PY-NLS, similarly to the previously discussed effect of P288S/A in A1.

### Disease associate mutations in hnRNPDL-2

**Figure 4.**
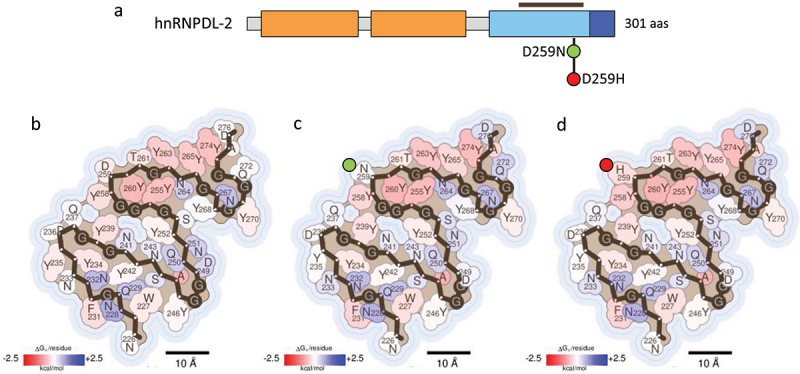
Impact of hnRNPDL-2 disease-associated mutations on fibril stability. (a) domain organization of hnRNPDL-2. The residues covered by the amyloid core in the cryo-EM hnRNPDL-2 fibril structure (PDB 7ZIR) [25] are indicated with a brown bar. (b-d) stabilization energy maps of the amyloid fibrils formed by the WT (b) and their corresponding disease-associated mutants D259N (c) and D259H (d). The structures are colored according to the energy values, as described in figure 2. In (a-d), the nature of the disease-associated mutations is indicated with green (stabilizing) or red (destabilizing) filled circles.

hnRNPDL is a highly conserved DNA- and RNA-binding hnRNP protein involved in mRNA biogenesis and alternative splicing regulation [11,45]. This protein exhibits a typical hnRNP composition, comprising two globular RRMs followed by a C-terminal LCD (aa 201–285), which contains the PY-NLS [10,25] Figure 4(a). Notably, alternative splicing renders three hnRNPDL isoforms with different aggregation and LLPS behaviours. hnRNPDL-2 (hereafter referred to as DL-2) is the predominant variant in human tissues and the only isoform capable of forming functional amyloid fibrils able of binding ssDNA/RNA [10]. The cryo-EM structure of the functional fibrils formed by full-length DL-2 was recently determined by García-Pardo and co-workers [25]. Their study provided insights into the overall arrangement of the RRMs within the fibril, revealing that they form a solenoidal coat surrounding the amyloid core. Contrary to what is observed in A1 and A2 proteins, the PY-NLS of DL-2 does not contribute to the formation of its amyloid core, highlighting distinct structural features specific to DL-2.

Missense mutations in DL-2 cause LGMD D3, a rare human myopathy driven by the impaired fibrillation of DL-2 mutant proteins. LGMD D3-associated mutations change the highly conserved D259 to either Asn or His. Similar to A1 and A2 analogous Asp mutations, D259N/H are located in DL-2 LCD, mapping into the amyloid core of the fibrils.

Energetic calculations of the D259N/H mutations on DL-2’s amyloid structure (PDB 7ZIR) [25] Figure 4(b-d) suggest that the D259N mutation slightly stabilizes the fibrils, resembling to Asp substitutions in other hnRNPs Table 1 and Figure 4(c). Conversely, the D259H mutation is predicted to be destabilizing Table 1 and Figure 4(d). However, neither mutation appears to lead to the formation of steric zippers, suggesting that these alterations may have a limited impact on fibril stability and might not necessarily induce disease through enhanced aggregation or increased fibril stability [25]. Supporting this hypothesis, ultracentrifugation studies of WT and mutant aggregates revealed that D259N/H proteins exhibit reduced amyloidogenicity compared to the WT protein. The resulting fibrils were observed to be shorter and less organized [25]. This finding aligns with the absence of aggregates in the muscular tissues of patients, suggesting a loss-of-function mechanism where mutant proteins are inefficient at forming functional amyloid structures in the affected muscle [25,46].

### Disease-associated mutations in TDP-43

**Figure 5.**
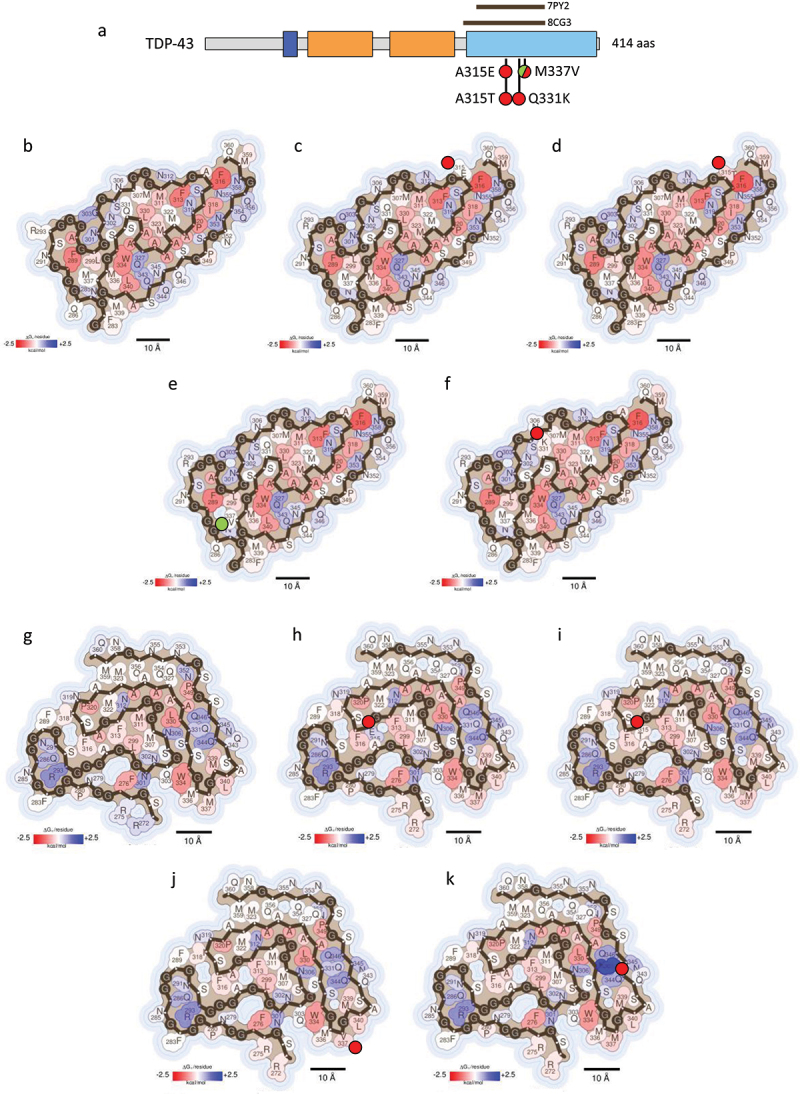
Impact of TDP-43 disease-associated mutations on fibril stability. (a) domain organization of TDP-43. The residues encompassed by the amyloid core of two *ex vivo* fibril structures of TDP-43, derived from patients with ALS-FTLD (PDB 7PY2) [27] and type A FTLD-TDP (PDB 8CG3) [28], are indicated with brown bars. b-k) stabilization energy maps of the ALS-FTLD (b-f) and type A FTLD-TDP (g-k) amyloid fibrils. The structures of the WT (b and g) and their corresponding disease-associated mutants A315E (c and h), A315T (d and i), M337V (e and j) and Q331K (f and k) are shown. The structures are colored according to the energy values, as described in figure 2. In a-k), the nature of the disease-associated mutations is indicated with green (stabilizing) or red (destabilizing) filled circles.

TDP-43 is a ubiquitously expressed hnRNP involved in multiple cellular processes, particularly in various stages of RNA metabolism. TDP-43 features a characteristic modular architecture, including an N-terminal domain containing the NLS, two RRMs, and a C-terminal LCD (aa 274–414) [7,9,47] Figure 5(a). While the N-terminal region of TDP-43 is crucial for its dimerization and RNA splicing activity, the LCD of this protein significantly influences its solubility and plays an important role in regulating its recruitment into SGs and its self-assembly into both functional and pathological aggregates [9,10,33].

The presence of inclusions containing full-length and fragmented TDP-43 is considered the main histopathological hallmark of ALS, FTLD and other neurodegenerative conditions, collectively known as TDP-43 proteinopathies [22,28,29]. However, this pathological signature is not exclusive to ALS and FTLD, as TDP-43 inclusions have also been identified in the brains of patients with other neurodegenerative disorders, including AD and PD [33,37].

Recently, Arseni and colleagues determined the cryo-EM structures of TDP-43 amyloid fibrils extracted from the frontal and motor cortices of patients with ALS-FTLD [27] and type A FTLD-TDP [28]. These seminal studies showed that each disease subtype was characterized by distinct polymorphic amyloid fibril structures, suggesting a strong association between polymorphism and disease. Thus, distinct mutations in TDP-43 can give rise to different fibril structures, impacting the disease phenotype [21]. In addition, abnormal post-translational modification (PTM) patterns, particularly increased phosphorylation in mutant proteins, have been implicated in promoting more stable and irreversible TDP-43 aggregation [34,35,48].

Over 40 point mutations in TDP-43 have been described so far, with nearly all of them located in the LCD and associated with disease [7–9]. These mutations exhibit diverse effects on the protein’s aggregation and LLPS behaviour. In this review, we will focus on describing four of the most prevalent and extensively studied mutations within the LCD of TDP-43 Table 1 and Figure 5(b-k). Specifically, the amino acid substitutions A315E, A315T and M337V in TDP-43 are mutations linked to familial ALS, while Q331K is associated to the development of sporadic ALS [49]. Despite the different nature of these mutations, all of them have been reported to display a similar effect on TDP-43 aggregation, enhancing filament formation [22,33,35,48–50] and seeding capacity [22,50]. In addition, these mutations lead to equivalent patterns of cytoplasmic mislocalization of TDP-43 [34,49] and modify LLPS behaviour, resulting in less dynamic condensates [36,50]. This altered behaviour contributes to neuronal dysfunction and cell death by disrupting TDP-43’s normal functions [22,33,50]. Furthermore, these disease-associated mutants not only present modified self-assembly properties but also lose their protective function against DNA damage, which leads to an accumulation of DNA lesions and compromises genome integrity in ALS patients [51].

Although some authors have suggested that mutations in TDP-43 enhance fibril stability [34,37,47,49], resulting in the accumulation of irreversible aggregates [33,36], there is a lack of consensus in this regard. To shed light on the potential role of these mutations in TDP-43 pathogenesis, we have conducted energy calculations using two recently solved prototypical structures from patients with ALS-FTLD (PDB 7PY2) [27] Table 1 and Figure 5(b-f) or with type A FTLD-TDP (PDB 8CG3) [28] Table 1 and Figure 5(g-k). Our analysis indicates that nearly all the examined mutations are predicted to be destabilizing. The only exception is the M337V mutation, which appears to slightly increase the stability of the fibril structure derived from ALS-FTLD patients Table 1 and Figure 5(e).

Previous studies have reported an aberrant LLPS behaviour of TDP-43 condensates featuring the M337V or Q331K mutations [50]. Notably, both M337 and Q331 residues are situated within the amyloid core of the TDP-43 structure derived from ALS with FTLD patients. These residues participate in mediating ß-sheet interactions that could be important for the dynamics of TDP-43 aggregation Figure 5(b-f). Specifically, the Q331K substitution introduces an unbalanced charged group within the amyloid core structure, potentially explaining its predicted destabilizing effect Table 1 and Figure 5(f).

In contrast, the M337 residue appears exposed to the solvent in the amyloid structure associated with type A FTLD-TDP Figure 5(j). This positioning could account for the opposed energetic effect of the M337V mutation in the two analysed amyloid structures.

In the case of residue A315, its structural positioning varies in amyloid fibrils derived from different pathological contexts. Specifically, in amyloid fibrils associated with ALS-FTLD, A315 is exposed to the solvent Figure 5(c-d), whereas in the type A FTLD-TDP structure, it forms part of the amyloid core Figure 5(h-i). This structural context correlates with the more pronounced destabilizing effects observed for both the A315E and A315T mutations in the type A FTLD-TDP amyloid fibrils, as depicted in Figure 5. Furthermore, the *ex vivo* TDP-43 structure from ALS-FTLD cases revealed two additional densities adjacent to solvent-exposed residues R293 and A315, suggesting potential interaction sites. Previous research has indicated that single-stranded DNA (ssDNA) interactions with TDP-43 can facilitate the assembly of the WT protein into a hydrogel and trigger the irreversible precipitation of proteins associated with ALS [33,48]. Mutations such as A315E/T might interfere with these ssDNA interactions, potentially altering TDP-43 self-assembly. Ultimately, TDP-43 aggregation is influenced by PTMs, including phosphorylation [27,34]. The introduction of a threonine residue by the A315T mutation could create a new phosphorylation site, potentially impacting the assembly properties of TDP-43.

### Disease-associated mutations in FUS

**Figure 6.**
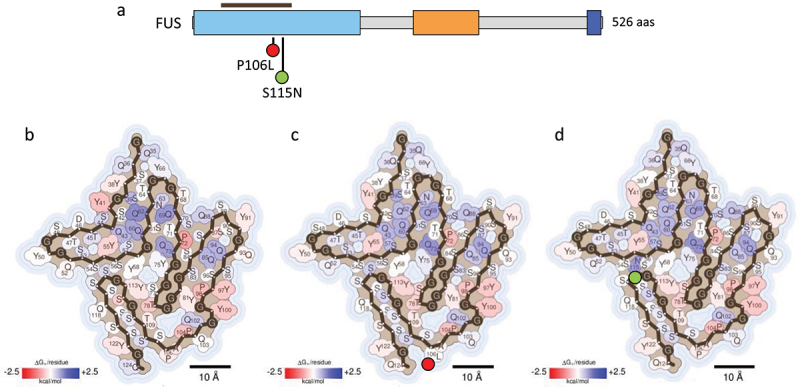
Impact of FUS disease-associated mutations on fibril stability. (a) domain organization of FUS. The residues covered by the amyloid core in the cryo-EM FUS fibril structure (PDB 7VQQ) [24] are indicated with a brown bar. (b-d) stabilization energy maps of the amyloid fibrils formed by the WT protein (b) and their corresponding disease-associated mutants P106L (c) and S115N (d). The structures are colored according to the energy values, as described in figure 2. In a-d), the nature of the disease-associated mutations is indicated with green (stabilizing) or red (destabilizing) filled circles.

FUS is a hnRNP that participates in various cellular processes, including DNA damage repair, transcription regulation, cell proliferation and RNA metabolism [7,9,38]. As depicted in Figure 6(a), FUS has a complex architecture composed of a single globular RRM domain, a N-terminal LCD (aa 1–214), multiple Glycine-rich regions and a C-terminal PY-NLS that mediate its nucleocytoplasmic shuttling [7,10,38]. Similar to TDP-43, the FUS LCD is an essential element that mediates protein-protein interactions within condensates [10]. Moreover, both FUS and TDP-43 share common functional and pathological features, including their association to neurodegeneration [38].

FUS was recognized as the primary component of TDP-43-negative cytoplasmic inclusions observed in rare ALS and FTLD cases, who presented FUS causative genetic mutations [9,38]. In contrast to TDP-43, most of the identified pathogenic mutations in FUS are located in the PY-NLS region [9,38]. Often these mutations affect the nuclear import of this protein, leading to its accumulation in the cytosol and giving rise to a gain of toxic function [4,7,9]. Moreover, different single-point mutations in the LCD of this protein have been associated to a conversion of functional and reversible liquid-like cytoplasmatic droplets into solid and more stable assemblies that accumulate as cytoplasmatic inclusions within degenerating motor neurons [9,10,38]. Despite extensive research, the pathogenic mechanisms underlying these mutations remain poorly understood.

In 2022, Sun and co-workers reported the cryo-EM fibrillar structure formed by the full-length FUS LCD fused to an N-terminal mCerulean tag [24]. This structure revealed a dominant fibril conformation displaying a unique serpentine fold comprising 10 individual β-strands spanning residues 34–124. We, therefore, assessed whether the disease-associated mutations targeting this region (*i.e* P106L and S115N) affect the stability of FUS amyloid fibrils Figure 6(b-d). As shown in Table 1 and Figure 6, energy calculations report destabilizing and stabilizing effects for P106L and S115N mutations, respectively. It is important to note that the P106L substitution adds a hydrophobic residue that cannot be easily accommodated into the amyloid structure, leaving the side chain of this new residue exposed the solvent, which may explain the decreased fibril stability. In contrast, the S115N mutation is likely to introduce additional stabilizing hydrogen bond interactions within the amyloid core, which is consistent with our energy calculations.

Previously, it has been reported that the interaction of FUS with specific RNAs modulates protein self-assembly and granule integrity [52]. In a similar context, single-point mutations in FUS have been associated to an altered RNA binding and enhanced FUS aggregation [53]. It is possible that the P106L mutation in FUS might exert comparable effects by altering the local residue environment and its interactions. Alternatively, this mutation might result in a different polymorph in which the newly introduced leucine residue resides at the solvent protected core of the fibril, stabilizing it through increased hydrophobic packing.

## Concluding remarks

RBPs form RNP complexes with RNA, playing an essential role in RNA metabolism and alternative splicing regulation [7–11]. A subset of these RBPs belongs to the hnRNPs protein family, whose functionality is intricately tied to their ability to transition between monomeric and amyloid-like states [54]. This conformational switch is primarily orchestrated by their LCD regions [55]. Notably, mutations within the LCD of hnRNPs have been associated with altered protein self-assembly and functionality, leading to a variety of neurodegenerative diseases, including ALS, FTD, MSP, AD, and PD. Despite significant progress in genetic research on these diseases, the precise pathogenic mechanisms of these mutations remain incompletely understood.

Recent advancements in cryo-EM have enabled the elucidation of the structures of amyloid fibrils formed by different hnRNPs, including A1, A2, DL-2, TDP-43, and FUS LCDs. These structures have shed light on the molecular interactions within amyloid cores and have revealed fundamental conformational differences between functional and pathologic amyloids.

In this review, we analyse the current literature addressing the structure of amyloid assemblies formed by hnRNPs and discuss how disease-associated mutations might impact their structure and function. We have systematically evaluated the energetic impact of prevalent mutations targeting the LCDs of these proteins. Contrary to the prevailing notion that mutations within the hnRNPs’ amyloid core invariably lead to more stable and irreversible aggregates, our calculations revealed a predominance of destabilizing effects induced by disease-related mutations in the analysed structures. However, we also observed a set of mutations causing a significant stabilizing effect, reinforcing intra- and inter-subunit interactions.

Our analysis highlighted specific trends for certain residue substitutions. For instance, mutations involving Asp residues frequently result in a stabilization of the amyloid fold. Conversely, substitutions involving Pro or Ala residues tend to promote destabilizing effects. It is now clear that a single protein sequence can give rise to distinct fibril polymorphs, each associated with a different disease condition. Similarly, the same mutation can induce distinct effects on different polymorphs, contingent upon the local environment of the affected residue. This is the case of the TDP-43 M337V mutation, which exerts divergent stabilizing or destabilizing influences depending on the specific TDP-43 amyloid structure being analysed.

Overall, our findings suggest that fibril stability alone is not the sole determinant of hnRNPs pathogenesis. Neither amyloid structure stabilization nor destabilization could be univocally associated with toxicity or altered function. Ultimately, the LCD mutations that we analysed argue that pathogenicity does not always correlate with amyloidogenic propensity.

## Data Availability

Data sharing does not apply to this article as no new data were created or analysed in this study.

## References

[1] Nizhnikov AA, Antonets KS, Bondarev SA, et al. Prions, amyloids, and RNA: pieces of a puzzle. Prion. 2016;10(3):182–206. doi: 10.1080/19336896.2016.1181253 27248002 PMC4981203

[2] Sawaya MR, Hughes MP, Rodriguez JA, et al. The expanding amyloid family: structure, stability, function, and pathogenesis. Cell. 2021;184(19):4857–4873. doi: 10.1016/j.cell.2021.08.013 34534463 PMC8772536

[3] Thompson MJ, Sievers SA, Karanicolas J, et al. The 3D profile method for identifying fibril-forming segments of proteins. Proc Natl Acad Sci. 2006;103(11):4074–4078. doi: 10.1073/pnas.0511295103 16537487 PMC1449648

[4] Kim HJ, Kim NC, Wang Y-D, et al. Mutations in prion-like domains in hnRNPA2B1 and hnRNPA1 cause multisystem proteinopathy and ALS. Nature. 2013;495(7442):467–473. doi: 10.1038/nature11922 23455423 PMC3756911

[5] Behbahanipour M, García-Pardo J, Ventura S. Decoding the role of coiled-coil motifs in human prion-like proteins. Prion. 2021;15(1):143–154. doi: 10.1080/19336896.2021.1961569 34428113 PMC8386614

[6] King OD, Gitler AD, Shorter J. The tip of the iceberg: RNA-binding proteins with prion-like domains in neurodegenerative disease. Brain Res. 2012;1462:61–80. doi:10.1016/j.brainres.2012.01.016 22445064 PMC3372647

[7] Song J. Molecular mechanisms of phase separation and amyloidosis of ALS/FTD-linked FUS and TDP-43. Aging Dis. 2023. doi: 10.14336/AD.2023.1118 PMC11346406 38029395

[8] Ocharán-Mercado A, Loaeza-Loaeza J, Castro-Coronel Y, et al. RNA-Binding Proteins: A Role in Neurotoxicity? Neurotox Res. 2023;41(6):681–697. doi: 10.1007/s12640-023-00669-w 37776476 PMC10682104

[9] Purice MD, Taylor JP. Linking hnRNP function to ALS and FTD pathology. Front Neurosci. 2018;12:326. doi:10.3389/fnins.2018.00326 29867335 PMC5962818

[10] Garcia-Pardo J, Ventura S. Cryo-EM structures of functional and pathological amyloid ribonucleoprotein assemblies. Trends Biochem Sci. 2023; 49 (2) 119–133. doi:10.1016/j.tibs.2023.10.005 37926650

[11] Batlle C, Ventura S. Prion-like domain disease-causing mutations and misregulation of alternative splicing relevance in limb-girdle muscular dystrophy (LGMD) 1G. Neural Regen Res. 2020;15(12):2239. doi: 10.4103/1673-5374.284988 32594036 PMC7749493

[12] Kemmerer K, Fischer S, Weigand JE. Auto- and cross-regulation of the hnRNPs D and DL. RNA. 2018;24(3):324–331. doi: 10.1261/rna.063420.117 29263134 PMC5824352

[13] Li Z, Wei H, Hu D, et al. Research progress on the structural and functional roles of hnRNPs in muscle development. Biomolecules. 2023;13(10):1434. doi: 10.3390/biom13101434 37892116 PMC10604023

[14] Scheres SHW. Amyloid structure determination in RELION-3.1. Acta Crystallogr D Struct Biol. 2020;76(2):94–101. 10.1107/S2059798319016577 32038040 PMC7008511

[15] Lövestam S, Scheres SHW High-throughput cryo-EM structure determination of amyloids. Faraday Discuss. 2022;240, 243–260. 10.1039/D2FD00034B 35913272 PMC9642048

[16] Thurber KR, Yin Y, Tycko R. Automated picking of amyloid fibrils from cryo-EM images for helical reconstruction with RELION. J Struct Biol. 2021;213(2):107736. doi: 10.1016/j.jsb.2021.107736 33831509 PMC8217313

[17] Sun Y, Zhao K, Xia W, et al. The nuclear localization sequence mediates hnRNPA1 amyloid fibril formation revealed by cryoEM structure. Nat Commun. 2020;11(1):6349. doi: 10.1038/s41467-020-20227-8 33311513 PMC7733464

[18] Lu J, Cao Q, Hughes MP, et al. CryoEM structure of the low-complexity domain of hnRNPA2 and its conversion to pathogenic amyloid. Nat Commun. 2020;11(1):4090. doi: 10.1038/s41467-020-17905-y 32796831 PMC7427792

[19] Lu J, Ge P, Sawaya MR, et al. Cryo-EM structures of the D290V mutant of the hnRNPA2 low-complexity domain suggests how D290V affects phase separation and aggregation. J Biol Chem. 2023; 300(2): 105531. doi:10.1016/j.jbc.2023.105531 38072051 PMC10844680

[20] Kumar ST, Nazarov S, Porta S, et al. Seeding the aggregation of TDP-43 requires post-fibrillization proteolytic cleavage. Nat Neurosci. 2023;26(6):983–996. doi: 10.1038/s41593-023-01341-4 37248338 PMC10244175

[21] Li Q, Babinchak WM, Surewicz WK. Cryo-EM structure of amyloid fibrils formed by the entire low complexity domain of TDP-43. Nat Commun. 2021;12(1):1620. doi: 10.1038/s41467-021-21912-y 33712624 PMC7955110

[22] Cao Q, Boyer DR, Sawaya MR, et al. Cryo-EM structures of four polymorphic TDP-43 amyloid cores. Nat Struct Mol Biol. 2019;26(7):619–627. doi: 10.1038/s41594-019-0248-4 31235914 PMC7047951

[23] Lee M, Ghosh U, Thurber KR, et al. Molecular structure and interactions within amyloid-like fibrils formed by a low-complexity protein sequence from FUS. Nat Commun. 2020;11(1):5735. doi: 10.1038/s41467-020-19512-3 33184287 PMC7665218

[24] Sun Y, Zhang S, Hu J, et al. Molecular structure of an amyloid fibril formed by FUS low-complexity domain. iScience. 2022;25(1):103701. doi: 10.1016/j.isci.2021.103701 35036880 PMC8749265

[25] Garcia-Pardo J, Bartolomé-Nafría A, Chaves-Sanjuan A, et al. Cryo-EM structure of hnRNPDL-2 fibrils, a functional amyloid associated with limb-girdle muscular dystrophy D3. Nat Commun. 2023;14(1):239. doi: 10.1038/s41467-023-35854-0 36646699 PMC9842712

[26] Sharma K, Banerjee S, Savran D, et al. Cryo-EM structure of the full-length hnRNPA1 amyloid fibril. J Mol Biol. 2023;435(18):168211. doi: 10.1016/j.jmb.2023.168211 37481159 PMC10530274

[27] Arseni D, Hasegawa M, Murzin AG, et al. Structure of pathological TDP-43 filaments from ALS with FTLD. Nature. 2022;601(7891):139–143. doi: 10.1038/s41586-021-04199-3 34880495 PMC7612255

[28] Arseni D, Chen R, Murzin AG, et al. TDP-43 forms amyloid filaments with a distinct fold in type a FTLD-TDP. Nature. 2023;620(7975):898–903. doi: 10.1038/s41586-023-06405-w 37532939 PMC10447236

[29] Beijer D, Kim HJ, Guo L, et al. Characterization of HNRNPA1 mutations defines diversity in pathogenic mechanisms and clinical presentation. JCI Insight. 2021;6(14). doi: 10.1172/jci.insight.148363 PMC8410042 34291734

[30] Gui X, Luo F, Li Y, et al. Structural basis for reversible amyloids of hnRNPA1 elucidates their role in stress granule assembly. Nat Commun. 2019;10(1):2006. doi: 10.1038/s41467-019-09902-7 31043593 PMC6494871

[31] Ryan VH, Perdikari TM, Naik MT, et al. Tyrosine phosphorylation regulates hnRNPA2 granule protein partitioning and reduces neurodegeneration. EMBO J. 2021;40(3):e105001. doi: 10.15252/embj.2020105001 33349959 PMC7849316

[32] Kim HJ, Mohassel P, Donkervoort S, et al. Heterozygous frameshift variants in HNRNPA2B1 cause early-onset oculopharyngeal muscular dystrophy. Nat Commun. 2022;13(1):2306. doi: 10.1038/s41467-022-30015-1 35484142 PMC9050844

[33] Lim L, Wei Y, Lu Y, et al. ALS-Causing mutations significantly perturb the self-assembly and interaction with Nucleic Acid of the intrinsically disordered Prion-like domain of TDP-43. PLoS Biol. 2016;14(1):e1002338. doi: 10.1371/journal.pbio.1002338 26735904 PMC4703307

[34] Guenther EL, Cao Q, Trinh H, et al. Atomic structures of TDP-43 LCD segments and insights into reversible or pathogenic aggregation. Nat Struct Mol Biol. 2018;25(6):463–471. doi: 10.1038/s41594-018-0064-2 29786080 PMC5990464

[35] Chien H-M, Lee C-C, Huang JJ-T. The different faces of the TDP-43 Low-complexity domain: the formation of liquid droplets and amyloid fibrils. IJMS. 2021;22(15):8213. 10.3390/ijms22158213 34360978 PMC8348237

[36] Vishal SS, Wijegunawardana D, Salaikumaran MR, et al. Sequence determinants of TDP-43 Ribonucleoprotein condensate formation and axonal transport in neurons. Front Cell Dev Biol. 2022;10:876893. doi:10.3389/fcell.2022.876893 35646935 PMC9133736

[37] Ling S-C, Albuquerque CP, Han JS, et al. ALS-associated mutations in TDP-43 increase its stability and promote TDP-43 complexes with FUS/TLS. Proc Natl Acad Sci. 2010;107(30):13318–13323. doi: 10.1073/pnas.1008227107 20624952 PMC2922163

[38] Deng H, Gao K, Jankovic J. The role of FUS gene variants in neurodegenerative diseases. Nat Rev Neurol. 2014;10(6):337–348. doi: 10.1038/nrneurol.2014.78 24840975

[39] Van Blitterswijk M, van Es MA, Hennekam EAM, et al. Evidence for an oligogenic basis of amyotrophic lateral sclerosis. Hum Mol Genet. 2012;21(17):3776–3784. doi: 10.1093/hmg/dds199 22645277

[40] Shishkin S, Kovalev L, Pashintseva N, et al. Heterogeneous nuclear ribonucleoproteins involved in the functioning of telomeres in malignant cells. IJMS. 2019;20(3):745. doi: 10.3390/ijms20030745 30744200 PMC6387250

[41] Tsoi PS, Quan MD, Choi K-J, et al. Electrostatic modulation of hnRNPA1 low‐complexity domain liquid–liquid phase separation and aggregation. Protein Sci. 2021;30(7):1408–1417. doi: 10.1002/pro.4108 33982369 PMC8197420

[42] Rohl CA, Strauss CEM, Misura KMS, et al. Protein structure prediction using Rosetta. Methods Enzymol. 2004;383:66–93. doi: 10.1016/S0076-6879(04)83004-0 15063647

[43] Murray DT, Zhou X, Kato M, et al. Structural characterization of the D290V mutation site in hnRNPA2 low-complexity–domain polymers. Proc Natl Acad Sci. 2018;115(42). doi: 10.1073/pnas.1806174115 PMC6196502 30279180

[44] Ryan VH, Dignon GL, Zerze GH, et al. Mechanistic view of hnRNPA2 low-complexity domain structure, interactions, and phase separation altered by mutation and arginine methylation. Mol Cell. 2018;69(3):465–479.e7. doi: 10.1016/j.molcel.2017.12.022 29358076 PMC5801700

[45] Li RZ, Hou J, Wei Y, et al. hnRNPDL extensively regulates transcription and alternative splicing. Gene. 2019;687:125–134. doi: 10.1016/j.gene.2018.11.026 30447347

[46] Vieira NM, Naslavsky MS, Licinio L, et al. A defect in the RNA-processing protein HNRPDL causes limb-girdle muscular dystrophy 1G (LGMD1G). Hum Mol Genet. 2014;23(15):4103–4110. doi: 10.1093/hmg/ddu127 24647604

[47] Gopal PP, Nirschl JJ, Klinman E, et al. Amyotrophic lateral sclerosis-linked mutations increase the viscosity of liquid-like TDP-43 RNP granules in neurons. Proc Natl Acad Sci. 114, (2017). 12 10.1073/pnas.1614462114 PMC5373408 28265061

[48] Zhu L, Xu M, Yang M, et al. An ALS-mutant TDP-43 neurotoxic peptide adopts an anti-parallel β-structure and induces TDP-43 redistribution. Hum Mol Genet. 2014;23(25):6863–6877. doi: 10.1093/hmg/ddu409 25113748 PMC4245047

[49] Prasad A, Bharathi V, Sivalingam V, et al. Molecular mechanisms of TDP-43 misfolding and pathology in amyotrophic lateral sclerosis. Front Mol Neurosci. 2019;12:25. doi:10.3389/fnmol.2019.00025 30837838 PMC6382748

[50] Koehler LC, Grese ZR, Bastos ACS, et al. TDP-43 oligomerization and phase separation properties are necessary for autoregulation. Front Neurosci. 2022;16:818655. doi: 10.3389/fnins.2022.818655 35495061 PMC9048411

[51] Konopka A, Whelan DR, Jamali MS, et al. Impaired NHEJ repair in amyotrophic lateral sclerosis is associated with TDP-43 mutations. Mol Neurodegener. 2020;15(1):51. doi: 10.1186/s13024-020-00386-4 32907630 PMC7488163

[52] Shelkovnikova TA, Robinson HK, Southcombe JA, et al. Multistep process of FUS aggregation in the cell cytoplasm involves RNA-dependent and RNA-independent mechanisms. Hum Mol Genet. 2014;23(19):5211–5226. doi: 10.1093/hmg/ddu243 24842888 PMC4159159

[53] Niaki AG, Sarkar J, Cai X, et al. Loss of dynamic RNA interaction and aberrant phase separation induced by two distinct types of ALS/FTD-Linked FUS mutations. Molecular Cell. 2020;77(1):82–94.e4. doi: 10.1016/j.molcel.2019.09.022 31630970 PMC6943187

[54] Geuens T, Bouhy D, Timmerman V. The hnRNP family: insights into their role in health and disease. Hum Genet. 2016;135(8):851–867. doi: 10.1007/s00439-016-1683-5 27215579 PMC4947485

[55] Molliex A, Temirov J, Lee J, et al. Phase separation by low complexity domains promotes stress granule assembly and drives pathological fibrillization. Cell. 2015;163(1):123–133. doi: 10.1016/j.cell.2015.09.015 26406374 PMC5149108

